# Modelling across Multiple Scales to Design Biopolymer Membranes for Sustainable Gas Separations: 1—Atomistic Approach

**DOI:** 10.3390/polym15071805

**Published:** 2023-04-06

**Authors:** Kseniya Papchenko, Eleonora Ricci, Maria Grazia De Angelis

**Affiliations:** 1 Institute for Materials and Processes, School of Engineering, University of Edinburgh, Sanderson Building, Robert Stevenson Road, Edinburgh EH9 3FB, UK; 2 Department of Civil, Chemical Environmental and Materials Engineering, DICAM, University of Bologna, Via Terracini 28, 40131 Bologna, Italy; 3 National Interuniversity Consortium of Materials Science and Technology INSTM, Via G. Giusti, 58100 Firenze, Italy

**Keywords:** gas separation, biopolymers, molecular modelling

## Abstract

In this work, we assessed the CO_2_ and CH_4_ sorption and transport in copolymers of 3-hydroxybutyrate and 3-hydroxyvalerate (PHBV), which showed good CO_2_ capture potential in our previous papers, thanks to their good solubility–selectivity, and are potential biodegradable alternatives to standard membrane-separation materials. Experimental tests were carried out on a commercial material containing 8% of 3-hydroxyvalerate (HV), while molecular modelling was used to screen the performance of the copolymers across the entire composition range by simulating structures with 0%, 8%, 60%, and 100% HV, with the aim to provide a guide for the selection of the membrane material. The polymers were simulated using molecular dynamics (MD) models and validated against experimental density, solubility parameters, and X-ray diffraction. The CO_2_/CH_4_ solubility–selectivity predicted by the Widom insertion method is in good agreement with experimental data, while the diffusivity–selectivity obtained via mean square displacement is somewhat overestimated. Overall, simulations indicate promising behaviour for the homopolymer containing 100% of HV. In part 2 of this series of papers, we will investigate the same biomaterials using a macroscopic model for polymers and compare the accuracy and performance of the two approaches.

## 1. Introduction

Membrane-based separation of gaseous mixtures is recognised as a low-energy and low-carbon alternative to traditional separation techniques [1,2,3,4]. The use of membranes can reduce the overall footprint of chemical processes, but most polymeric materials used for this purpose cannot be considered sustainable since they are fossil-based. Most industrial applications require a full replacement of the membrane module every three to five years [5,6], and recent works focused on finding renewable alternatives for membrane materials, such as bio-based and biodegradable polymers [2,7,8].

Polymers of the poly(hydroxyalkanoate)s (PHAs) family are attractive, as their origin is fully renewable, and biodegradability can be achieved in many environments [9,10]. Indeed, PHAs are linear polyesters synthesised naturally by several microorganisms as an energy reserve, while also well-established chemical routes for controlled PHA synthesis were developed [9,11,12,13]. Poly(3-hydroxybutyrate) (PHB), poly(3-hydroxyvalerate) (PHV), and their copolymers are currently the most studied and produced bio-polyesters, as the application range of such materials appears to be quite broad [14,15,16]. At the same time, the experimental characterisation of fluid transport properties, to this day, was reported only for a low number of gases in a small subset of PHAs [17,18,19].

Despite the attractiveness of such materials, several issues might delay their commercialisation, such as the difficulty in finding an optimal formulation which has good overall properties. On the other hand, a systematic experimental study is expensive and time-consuming, as there are many possible candidates. Computational approaches that can provide structure-property relationships faster need to be considered to reduce the time-to-market of biopolymers like the ones inspected here.

In this work, we focus on transport properties, specifically gas solubility, diffusivity, and permeability, for which different modelling techniques have been proposed in the literature and are readily-available to use [20]. In particular, molecular dynamics (MD) simulations have been proven efficient in predicting a range of polymer properties [21,22,23]. Recently, different groups applied MD to study new possible applications for PHAs [24] and to successfully investigate the thermal and mechanical properties of such materials [25,26,27].

In this work, an analogous approach was used to explore the gas transport in PHAs, with the aim of using them as sustainable membranes for gas separation. MD simulations were conducted to predict the CO_2_/CH_4_ separation performance of PHBV copolymers with different compositions, which is of interest for CO_2_ capture, biomethane purification and negative-emission processes. The computational screening of different structures to identify the most promising ones allows for the reduction of tests and guides future experimental efforts. The polymer models were validated using data coming from the literature [28,29,30,31,32,33], while for the gas-polymer mixtures, specific gas-sorption tests were conducted in this work.

## 2. Materials and Methods

### 2.1. Experimental

#### Materials

**Figure 1:**
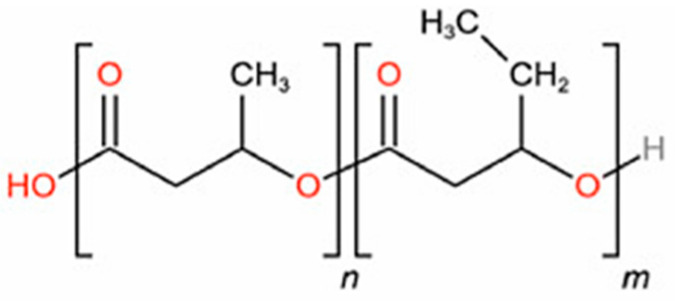
Chemical structure of poly(3-hydroxybutyrate-*co*-3-hydroxyvalerate) PHBV used in this work.

Bacterial poly(3-hydroxybutyrate-*co*-3-hydroxyvalerate) (PHBV), 8 mol% hydroxyvalerate (HV) units, was purchased from Sigma-Aldrich (St. Louis, MO, USA) in the form of film and used as received. The chemical structure of the biopolymer used in this study is shown in Figure 1. The thickness was measured to be 19 ± 1 µm. The crystallinity was not provided by the producers and was determined to be equal to 42 ± 1% through DSC analysis, as described in Section S1 of the Supporting Information (SI) [19]. Gases used for sorption tests, namely methane (CH_4_) and carbon dioxide (CO_2_), were purchased from Fluido Tecnica (Campi Bisenzio, FI, Italy) with purities ≥ 99.5%. The density of the semi-crystalline membrane was assumed equal to 1.214 g·cm^−3^, as reported by Mitomo et al. for an 8% HV copolymer [28].

### 2.2. Gas Solubility and Diffusivity Measurements

Solubility and diffusivity of CH_4_ and CO_2_ in PHBV were determined at 35 °C at pressures up to 10 bar in a pressure-decay apparatus [34]. The measurements were repeated at least twice for each gas. The full equipment set-up is well-represented and described elsewhere [35,36]. In this technique, a known amount of gas is fed into the sample chamber and the mass uptake is evaluated by measuring the pressure decrease over time. At equilibrium, the pressure becomes constant, as does the amount of gas absorbed by the membrane; after such values are recorded, a new pressure is imposed, and the procedure is repeated in a similar manner.

Sorption isotherms are constructed by connecting the equilibrium concentration values of the penetrant molecule in the polymer at each experimental pressure. The solubility coefficient S is the ratio between the concentration of the gas in the sample, c, and the pressure, p, as follows:

(1) $$S=\frac{c}{p}$$

Dense homogeneous matrices at temperatures above the glass transition, $T_{g}$, such as the ones inspected here, generally follow Henry’s law, which describes a linear correlation between the gas uptake and the equilibrium pressure, so that S is constant with pressure. In each sorption step, the penetrant diffusivity in the film, D, can be evaluated from the sorption kinetics by considering Fickian diffusion and the variation of interfacial concentration during the experiment, as reported in the literature [35,37,38] and shown in Section S2 of the SI.

The performance of the membrane in gas separation is usually evaluated by the permeability, P, proportional to the molar flux of the gas across the material, and the selectivity, α, which is equal to the ratio between gas permeabilities. Under the assumption of the validity of the solution diffusion model [39], the permeability coefficient can be split into the product of the diffusion coefficient, $D_{i}$, and the solubility coefficient, $S_{i}$, as follows:

(2) $$P_{i}=D_{i}S_{i}$$

Under the same assumptions, the ideal selectivity between gas *i* and gas *j*, $\alpha_{ij}$, becomes the ratio of pure-gas permeabilities, and can be split into the product of the ideal diffusivity–selectivity, $\alpha_{ij}^{D}$, and the ideal solubility–selectivity, $\alpha_{ij}^{S}$:

(3) $$\alpha_{ij}=\frac{P_{i}}{P_{j}}=\frac{D_{i}S_{i}}{D_{j}S_{j}}=\alpha_{ij}^{D}\alpha_{ij}^{S}$$

### 2.3. MD Simulations

Eight different PHBV copolymers were considered for density and solubility parameter estimation with 0, 8, 16, 24, 40, 60, 80, and 100 mol% of HV units, which in the following will be referred to as PHBV0, PHBV8, …, and PHBV100.

Monodispersed, amorphous polymeric melts at different compositions were generated using the Amorphous Builder plugin of the Materials and Processes Simulations (MAPS) software [40,41]. All systems were generated at 600 K. First, an energy minimization and short equilibration in the NVT (isothermal) and NPT (isobaric–isothermal) ensemble at 600 K was performed. Such equilibration allows to eliminate the overlaps that could have been introduced during the initial configuration generation and obtain a homogeneous density throughout the system. Then, a decreasing temperature ramp of 50 K/ns in the NPT ensemble was applied in order to reach the target value of 298 K, allowing the system to change the density according to the change in temperature imposed. Slower cooling rates were tested as well, and no appreciable difference in results was observed. Each system consisted of 5 chains of 150 monomers each, leading to molecular weight in the range between ~13,000 and 15,000 g/mol, number of atoms between ~9000 and 11,000, and box size between ~45 and 50 Å. For every system, three independent initial configurations were generated and simulated, allowing the extraction of the average value and standard deviation for each property.

Simulations were performed in full atomistic detail during all equilibration and production runs using the LAMMPS package (3 March 2020 stable release) [42,43]. The polymer consistent force field (PCFF) [44] was used for polymers, methane, and polymer-CH_4_ systems, while the COMPASS force field [45] was used to describe CO_2_ and its interactions with the polymer system.

All systems were simulated with periodic boundary conditions, and the cut-off for Lennard-Jones potential and Coulombic interactions was set to 12 Å as the maximum value above which the box energy no longer changes. In order to account for the long-range van der Waals interactions, tail corrections were included, while long-range electrostatics were computed with a particle–particle particle–mesh (pppm) method (relative error in forces calculations set to 10^−6^) [46]. Nosé–Hoover thermostat and barostat with dumping parameters of 100 fs and 500 fs were used for temperature and pressure control, respectively [47]. A timestep of 1 fs was adopted for runs in the NVT and NPT ensembles, while a shorter timestep of 0.5 fs was used for NVE (constant-energy) runs.

After the initial configurations were generated and cooled to 298 K, a short equilibration in the NVT ensemble was performed, and production NPT runs of 10 ns were performed at 298 K and 1 bar in order to extract average values of density and cohesive energy and compare them with data found in the literature. The systems were then heated to 308 K at 50 K/ns in the NPT ensemble, and NPT runs of 10 ns at 308 K and 1 bar were performed.

As the transport-properties analysis is computationally more expensive with respect to other properties retrieved in this work, we investigated such properties in a lower number of copolymers. The last 5 ns of the trajectories on 4 copolymers, namely PHBV0, PHBV8, PHBV60, and PHBV100 systems, were used to perform the Widom test particle insertions [48] in order to calculate the excess chemical potential of CO_2_ and CH_4_ at infinite dilution, as described elsewhere [23,49], and from that the solubility. Although several methods exist to simulate the solubility in polymers, for which we address the reader to competent and extended reviews [20,50], the Widom insertion method is deemed optimal for the low-concentration sorption of small molecules, as is the case inspected here.

A total of 10^6^ insertions per gas per pure polymer system was sufficient for the convergence of the value of the solubility coefficient, calculated as follows:

(4) $$\frac{1}{S_{i}}=\frac{\rho RT}{M_{i}}\underset{x_{i}\to 0}{\lim}\left[exp\left(-\frac{\mu_{i}^{ex}}{RT}\right)\right]$$

where ρ is the density of the pure polymer system, $M_{i}$ is the molar mass of CO_2_ or CH_4_, and $\mu_{i}^{ex}$ is the excess chemical potential of CO_2_ or CH_4_ at infinite dilution.

The solubility coefficient obtained from the Widom analysis and averaged over three configurations for each system was used to calculate the corresponding number of gas molecules to insert into each system at atmospheric pressure. The calculation was done under the assumption of a quasi-constant solubility coefficient at temperatures higher than the glass transition (T_g_) in PHBV, as confirmed by previous experimental work [19]. After the energy minimization, a short NVT run, a 10 ns NPT run, and a second 1 ns NVT run were performed for equilibration. Afterwards, 50 to 100 ns production runs in the NVE ensemble were performed in order to extract the average mean square displacements (MSDs) of gas molecules, to calculate the self-diffusion coefficients through Einstein’s relation, as follows [47,51]:

(5) $$D=\underset{t\to \infty }{\lim}\frac{1}{6t}\langle {\left|r\left(t\right)-r\left(0\right)\right|}^{2}\rangle$$

where r(t) and r(0) are the positions of the centre of mass of the gas molecule at time t and at the initial time t=0, respectively. Self-diffusivities are a good approximation of binary diffusivities in the case of an infinitely dilute system or when the sorption isotherm is linear [52]. The NVE ensemble is considered the most suitable to study the dynamic properties of the system, as no thermostat or barostat is used in such integration and thus no external influence is imposed on the system [53]. A multiple time origin scheme was considered in the calculation, and the Fickian regime of MSDs was reached in all cases. A representative example of diffusion coefficient calculation from MD simulations is shown in Section S3 of the SI. The NVE ensemble was also used to calculate the radial distribution functions, g(r), to evaluate the intermolecular interactions between gas molecules and the four polymeric structures, as described elsewhere [47]. In order to visualize the structural difference between the polymeric matrices, short NVE simulations were performed after production NPT runs at 308K and 1 bar, and short NVT equilibration runs. The static structure factor, S(q), was then calculated from the radial distribution functions of all atom types pairs and the atomic-form factors, $f_{i}(q)$ [54], as described elsewhere [23]. The peaks at a specific magnitude of the wave vector, q, can be compared to the peaks at a specific scattering angle in X-ray scattering patterns.

**Figure 2:**
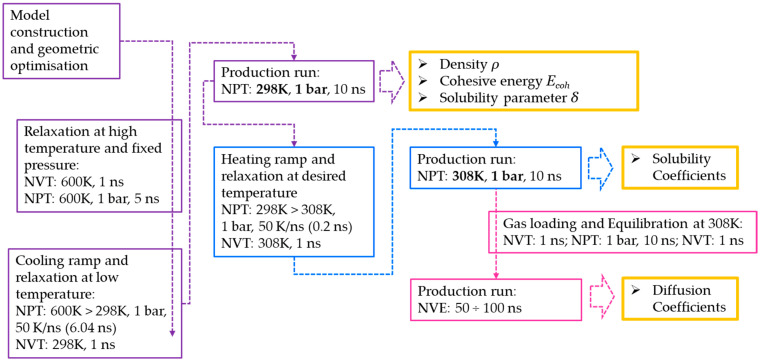
Simulation workflow.

The complete schematic of the workflow used in this work is shown in Figure 2.

Gas solubility and diffusivity values obtained from simulations were compared to the experimental values from this and previous work [19]. It is important to point out that, while in the case of pure polymer properties such as density and solubility parameters amorphous state data were available for validation, the experimental gas transport tests were carried out on real semi-crystalline samples. Full atomistic simulations do not allow observation of any crystallization in typical simulation times and are representative only of the free amorphous domains. The crystalline phase, however, is impenetrable and does not contribute directly neither to gas sorption or to gas diffusion, so some rules of thumb can be used.

In the case of solubility, the experiments measure the total semi-crystalline value, $S_{i}^{sc}$, whereas the simulations yield the solubility of a hypothetical free-amorphous phase only, $S_{i}^{am}$ [50]. Assuming, as discussed above, that the crystalline domains have zero solubility, a simple rule can be used to compare experimental and simulated values:

(6) $$S_{i}^{sc}=S_{i}^{am}\left(1-X_{c}\right)$$

where $X_{c}$ is the degree of crystallinity.

The scaling of the diffusivity coefficient on the crystalline content of the sample is somehow less straightforward because the crystals are impermeable and lengthen the diffusive path, as the gas molecules have to circumvent crystals to go from one amorphous area to the other. Such an effect depends on the number of crystals present but also on their shape and distribution. Presumably, the effect of crystals on diffusion is larger than what would be predicted by an additive rule such as the one represented by Equation (6), as we foresee overestimates as high as one order of magnitude. It is expected, however, that crystallinity effects are independent of the gas type and that diffusivity–selectivity simulations could be more reliable. Therefore we will only put in direct comparison the simulated and experimental values of the diffusivity–selectivity, leaving aside the gases' individual diffusion coefficients.

## 3. Results and Discussion

### 3.1. Validation of the Molecular Polymer Model

**Figure 3:**
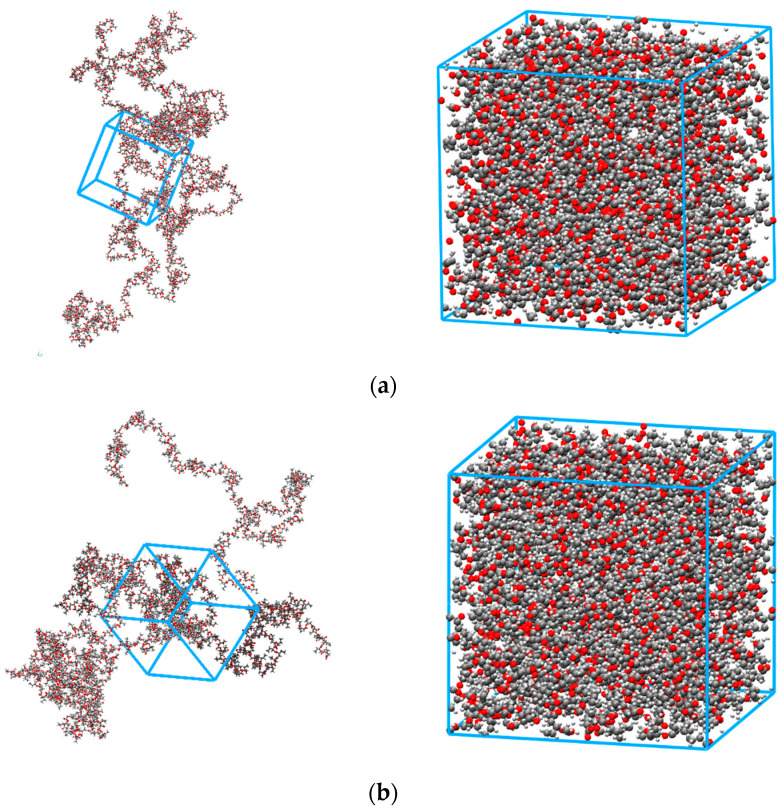
Snapshots of simulation boxes for PHBV0 (**a**) and PHBV100 (**b**) in expanded (**left**) and closed (**right**) form after equilibration.

When a specific force field is used to describe polymer behaviour, it is essential to confirm its capability to correctly reproduce volumetric and energetic parameters before predictions of the sorption and transport properties of different gases can be undertaken [20,21,22,23]. Figure 3 shows the snapshots of the equilibrated structures for two homopolymers as an example.

**Figure 4:**
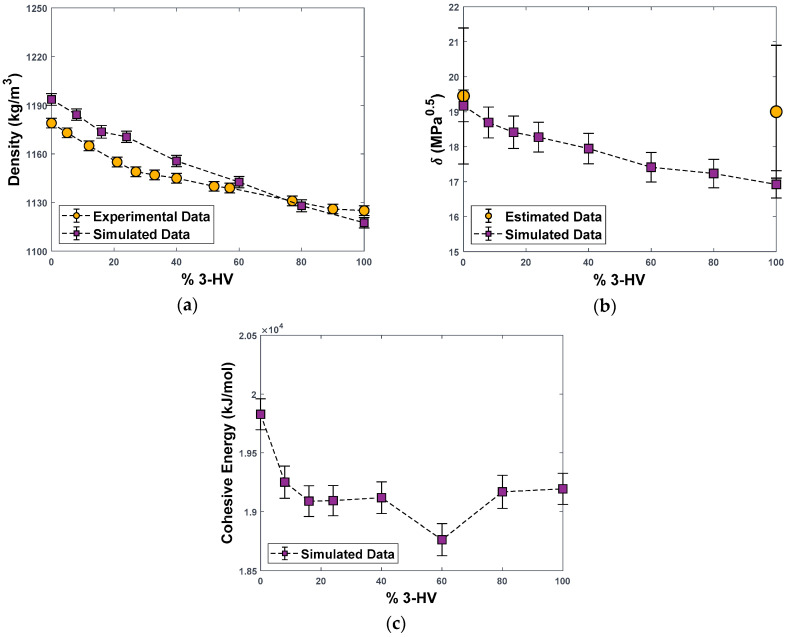
Density (**a**), Hildebrand solubility parameters (**b**), and Cohesive Energy (**c**) for PHBV copolymers as a function of HV molar percentage, as obtained from simulations in this work and experiments from the literature. Experimental density for amorphous PHBV from ref. [28]. Solubility parameter for PHBV0 and PHBV100 from [29,30].

It must be noticed that the polymer molecular models represent only the amorphous phase, as the current limits on the fully atomistic simulations considered here do not allow for observation of the polymer crystallization. For the pure polymer, however, the data used for model validation refer indeed to amorphous structures. In particular, PHBV copolymers were studied in the amorphous state by Mitomo et al. [28], who measured densities using the gradient column technique on samples obtained by melting at 185 °C for 2 min and immediately quenching in liquid nitrogen to avoid crystallization. As Figure 4a shows, the experimental density values, as well as their dependence on the amount of HV units present in the copolymer, are well-reproduced by the MD model, with deviations < 1% for all systems, except for PHBV24, where the deviation reaches 1.6%. The deviations were measured considering the linear decrease in the amorphous density, with different slopes at compositions lower and higher than 30 mol% of HV units, as described by Mitomo et al. [28]. In particular, one can see that the density decreases with increasing fraction of HV units in the polymer; this is due to the bulkier side group of the HV monomer, which inhibits the tight packing of the polymer chains.

The second quantity used to validate the molecular polymer model is an energetic one, namely Hildebrand’s solubility parameter, δ; a thermodynamic property that defines the miscibility and compatibility of the polymer with fluids. Such value is equal to the square root of the intermolecular cohesive energy of the system per unit volume, being higher for substances with strong internal attractive interactions such as hydrogen bonds. For low molecular weight liquids, this value can be estimated from the vaporization energy, while for polymers, it should be determined either by trial and error using experimental solubility data of the polymer in various solvents, or with group contribution methods, which are normally affected by errors of ±10%. In molecular simulations, as in the case of the present work, the intermolecular energy $E_{coh}$ may be evaluated as the difference between the sum of the intramolecular energy of every single polymer chain and the total potential energy of the simulation box [22]. The solubility parameter is then calculated as $\sqrt{\langle E_{coh}/V\rangle }$.

For pure PHB (PHBV0), the average literature values for δ vary between 19.45 [29] and 21.0 [30] MPa^0.5^, depending on the method used, while for PHBV100, values range between 18.6 and 19.4 MPa^0.5^ according to different group contribution methods [29]. The latter methods provide values for amorphous polymers, so they do not need correction before being compared to the ones obtained from atomistic simulations.

Figure 4b shows that the MD-simulated values fall within the uncertainty range estimated for the literature values (±10% [29]). The simulations also catch the qualitative trend of the solubility parameter decreasing with an increasing amount of HV units, which is due to a combination of the lower intrinsic polarity of the polymers richer in HV, as confirmed by decreasing cohesive energy (Figure 4c), and lower density.

In Figure S4 in the SI, we also reported values of the fractional free volume (FFV) for a probe of negligible size and the corresponding accessible surface area; both quantities agree with the trend of density and increase monotonically with the HV content. This is possibly due to the longer alkyl side group of the HV monomer, with respect to HB, which disrupts the packing of the polymer chains even in the amorphous state. The solubility parameter decreases with HV fraction due to the higher amount of non-polar groups brought by the HV monomer, that have a lower value of δ.

It is interesting to analyze the simulated models also in terms of their static structure factor *S(q)*. This can provide further validation of the generated structures, as the features at low values of q reflect intermolecular correlations in the bulk of the polymeric phase that can be compared directly to the peaks observed at low scattering angles in wide-angle X-ray diffraction patterns.

**Figure 5:**
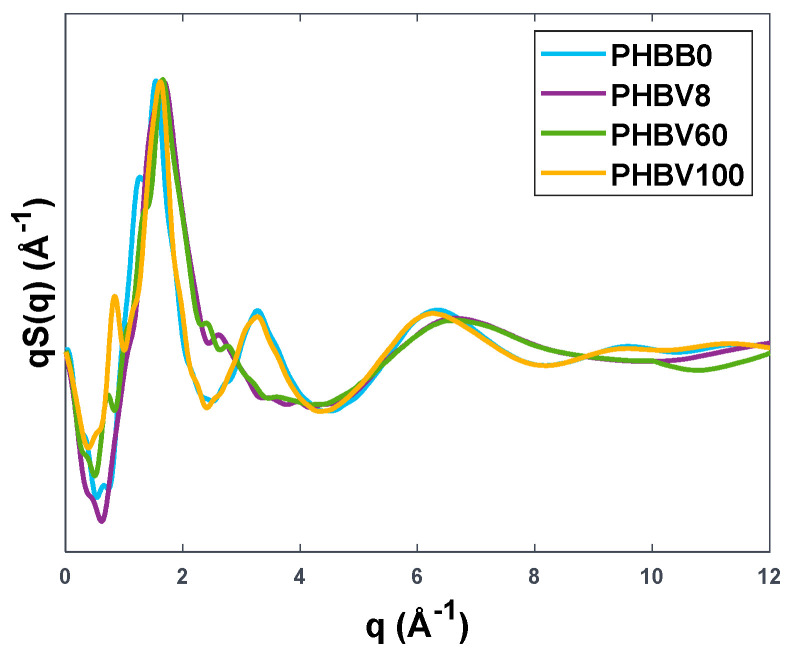
q—weighted static structure factor for several PHBV copolymers.

We analysed such features in the same polymers chosen for the study of transport properties, thus in PHBB0, PHBV8, PHBV60, and PHBV100. Figure 5 reports the structure factor weighted on the wave vector q. Interestingly, all four structures present quite a pronounced peak in a narrow range $q=1.63\pm 0.05{Å}^{-1}$, which in terms of scattering angle 2θ translates to $23.8\pm 0.8^{{}^{\circ}}$. This agrees well with the diffraction spectra of PHB and PHBV copolymers reported in the literature [31,32,33], where the amorphous peaks are found in the range of scattering angles $2\theta \approx 22-24^{{}^{\circ}}$. It can be evinced from Figure 5 that the structure of two homopolymers is very similar, with a narrower main peak with respect to the two copolymers. Such a result can be attributed to the fact that homopolymers have a more ordered and regular structure as opposed to random copolymers.

Given the satisfactory comparison with PHBV copolymer density, solubility parameter values, and structure features from the literature, the force field and simulation protocol adopted in this work were validated, and the same model was used to simulate the gas solubility and diffusivity.

### 3.2. Gas Separation Performance: Experimental Data and MD Simulations

In this work, we obtained direct data on CO_2_ and CH_4_ sorption and diffusion, through sorption tests on the PHBV8 copolymer, which can also be used to estimate permeability according to the solution diffusion model. Additional data come from a previous work where a PHBV25 copolymer was characterized [19], specifically with CO_2_ solubility, diffusivity and permeability values from sorption and permeation measurements and CH_4_ diffusivity and permeability values from permeation tests.

The measured values of CO_2_ and CH_4_ solubility coefficients, normalized for the crystallinity content, as well as the selectivities, are reported in Table S1 and later in the text.

The experimental solubility coefficient in PHBV8, appropriately scaled with the crystallinity fraction with Equation (6), is higher for CO_2_ than for CH_4_, which is due to the different condensability and the favourable energetic interactions of CO_2_ with the ester groups of the polymer. A similar behaviour was observed in PHBV25 in a previous work [19]. The resulting CO_2_/CH_4_ solubility–selectivity, evaluated on a molar basis, is equal to 5.0, while in the case of PHBV25, it was 13.9 [19].

Conversely, the diffusion coefficient of CO_2_ measured in PHBV8 is smaller than that of CH_4_, despite the fact that such a gas molecule has a larger kinetic diameter. Such behaviour results in a rather low value of diffusivity–selectivity for PHBV8 (0.6). The same value measured in PHBV25 was higher (1.9) but still rather limited considering the different molecular sizes of the two gases. We believe that the rather limited diffusivity–selectivity of CO_2_ in these matrices is due to the strong interactions that such penetrant has with the polyesters, which hinders its mobility, ultimately making the solubility the main driver of separation in PHBV matrices.

**Figure 6:**
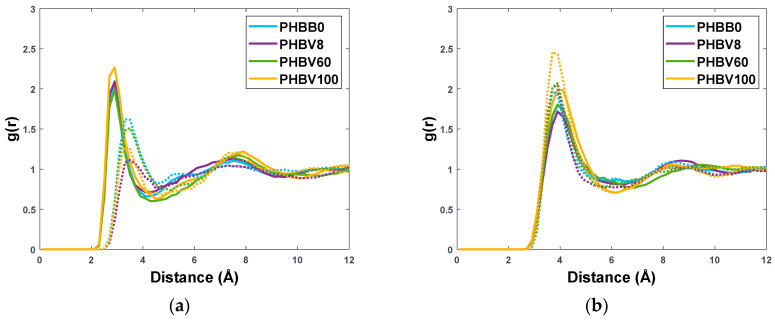
Radial distribution functions for intermolecular interactions between C−CO_2_ (straight line) or C−CH_4_ (dotted line) and Carbonyl O (**a**) or side chain C (**b**) in PHBB0, PHBV8, PHBV60, and PHBV100.

The above considerations can be co”robo’ated by computing the radial distribution functions, g(r), between polymeric atoms and the centre of mass of gas molecules, here represented by the carbon atom in CO_2_ and CH_4_, indicated as C-CO_2_ and C-CH_4,_ respectively. Complete results are reported in Section S4 and Figure S3 of the SI. Figure 6a shows that the peak relative to the interaction between C-CO_2_ and the oxygen atom on the carbonyl group of the copolymer is at a shorter distance with respect to the one relative to C-CH_4_. This does not happen systematically with other atom types, as it can be seen in the SI; therefore, it cannot be attributed solely to the smaller size of the CO_2_ molecule with respect to CH_4_, but rather to a specific interaction between CO_2_ molecules and ester groups of PHBVs. On the other hand, Figure 6b shows that both penetrant molecules are at the same distance with respect to carbons of the methyl and ethyl groups on the side chains, but the correlation is stronger in the case of CH_4_, as shown by the peak heights, suggesting a preferential positioning of the CH_4_ molecules towards the alkyl groups. However, the difference between CO_2_-related and CH_4_-related peaks is not as marked as in Figure 6a, which may justify the overall CO_2_-selective behaviour of such matrices. Another thing to notice is that, unlike the pure polymer molecular model, which shows very regular trends of density and solubility parameters with HV fraction, the gas-polymer models have RDF trends not monotonous with the content of HV. This is possibly due to the fact that FFV and δ have opposite trends with HV fraction, thus resulting in a more complex dependence. Indeed, as we will see it in the following, the behaviour of gas-polymer diffusivity, solubility, and selectivity will be a less regular function of the copolymer composition.

**Figure 7:**
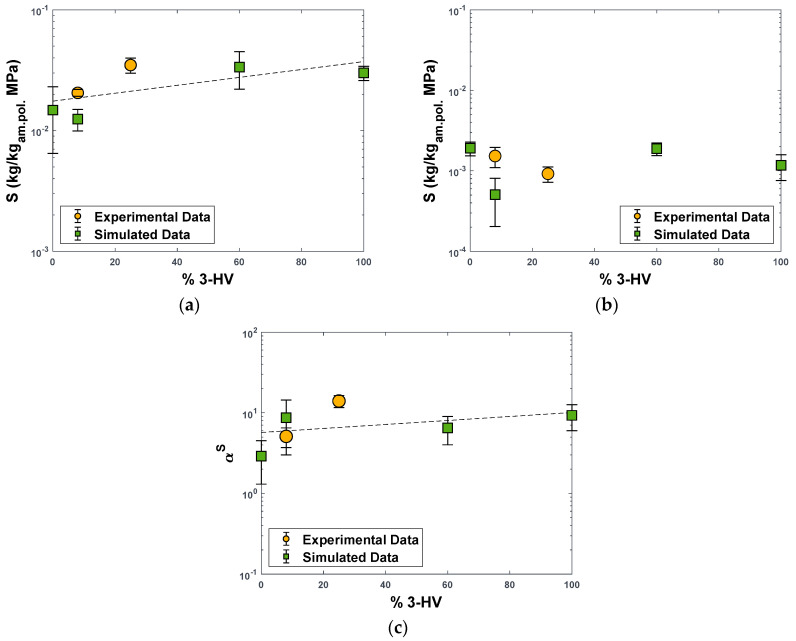
Solubility coefficient values for CO_2_ (**a**) and CH_4_ (**b**) and CO_2_/CH_4_ solubility−selectivity (**c**) in PHBV copolymers as a function of the molar concentration of HV units. Green squares: MD−simulated values. Yellow dots: experimental solubility data. For CO_2_ in PHBV25, data from [19]. For CH_4_ in PHBV25, data obtained as S=P/D in [19]. Data are expressed per kilograms of amorphous polymeric phase (Equation (6)). Dashed lines are linear interpolations of the simulated values.

In Table S1 and Figure 7, we reported the simulated solubility coefficients of CO_2_ and CH_4_ in PHBV0, PHBV8, PHBV60, and PHBV100, estimated through the Widom particle insertion method. It can be observed that the predicted value of gas solubility in the copolymer containing 8% of HV is 40% lower than the experimental counterpart in the case of CO_2_ and 67% lower in the case of CH_4_. However, such a discrepancy is in line with the current accuracy of the MD prediction of gas solubility in polymers. According to the specialist literature, a difference between measured and simulated values of *S* within a factor of 3 can be regarded as acceptable when approaches like the Widom particle insertion or the grand-canonical Monte Carlo (GCMC) are used [55,56,57,58,59,60].

The simulated solubility data at different compositions are characterised by a certain scattering, which is common for MD prediction of solubility in these structures, as discussed above, although interpolation of the values with a linear trend would indicate that CO_2_ solubility increases with the fraction of HV units (Figure 7a). The experimental trend observed on PHBV8 and PHBV25, and on PHBV with 8, 12, and 33 mol% HV from the literature [61] indicates that CO_2_ solubility increases with HV content, although a plateau is observed above 30% of HV [61], and polymer grades with HV fractions higher than 33% were not explored. Previous works attributed the higher solubility to the higher flexibility of copolymers containing more HV [61], which is consistent with our observed increase of FFV in materials richer in this monomer. On the other hand, the predicted CH_4_ solubility is constant or slightly decreases with an increasing amount of HV, as confirmed by the two experimental values obtained by our group on PHBV8 and PHBV25, but in this case, there are no independent measurements from other sources for comparison. As mentioned above, increasing the HV content has compounding effects on the binary gas-polymer systems, which show a less regular behaviour than the pure polymer alone.

The simulated value of CO_2_/CH_4_ solubility–selectivity for the PHBV8 copolymer is in good agreement with the experimental value, confirming that simulated selectivity factors are more reliable than individual transport coefficients (Figure 7c). The higher values of $\alpha_{S}$ are recorded for PHBV8 and PHBV100, which can be explained by the fact that those polymers show the highest interactions between CO_2_ and polymer carbonyls, according to the RDF spectra of Figure 6a. Overall, CO_2_ solubility and CO_2_/CH_4_ solubility–selectivity increase respectively by a factor of 2 and 3.2 going from PHBV0 to PHBV100 [62,63].

**Figure 8:**
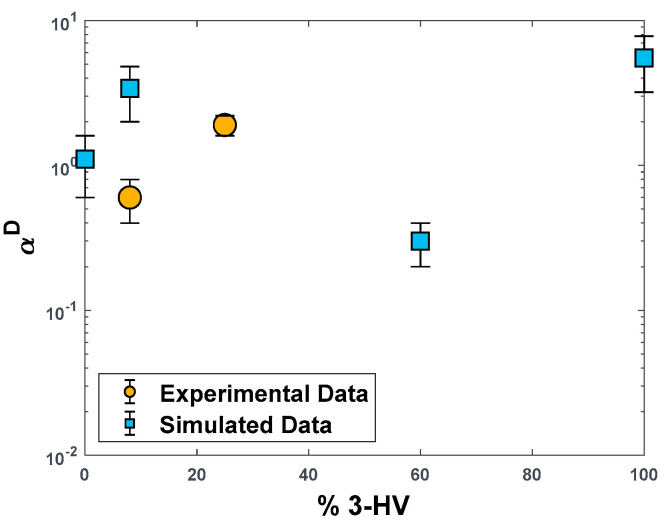
CO_2_/CH_4_ Diffusivity−selectivity values in PHBV copolymers as a function of the molar concentration of HV units. Blue squares: MD−simulated values on amorphous PHBV boxes. Yellow dots: experimental data obtained on semi−crystalline PHBV samples (diffusivity values are the timelag of permeation for the PHBV25 sample in [19]).

Diffusivity values were calculated from the mean square displacements of CO_2_ and CH_4_ molecules extracted from NVE trajectories. As the direct comparison between simulated values of diffusivity in the amorphous phase and measured ones in the semi-crystalline phase is not meaningful, because the first ones are significantly higher, we compared only the diffusivity–selectivity values in Figure 8 [56,58,59,64]. The model over-predicts this value by a factor of 5 in the case of PHBV8. A clear trend with composition is less visible in this case, although, as for all the other performance indicators, the value of diffusion selectivity for PHBV100 is the highest.

**Figure 9:**
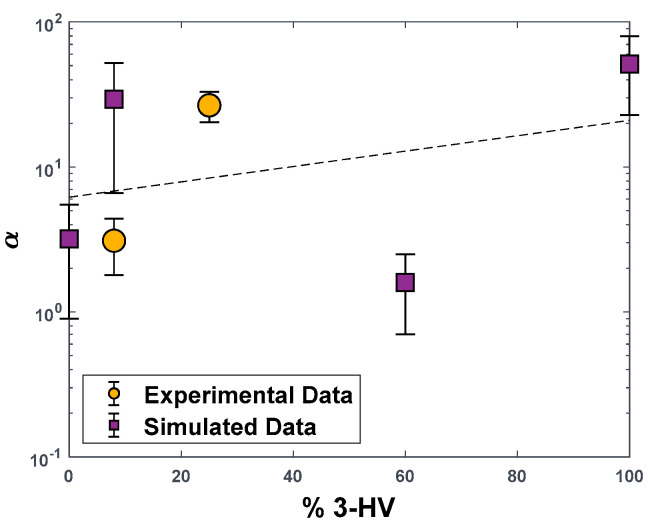
Permselectivity for the CO_2_/CH_4_ couple in PHBV copolymers as a function of the molar concentration of HV units. Violet squares: MD−simulated values on amorphous PHBV boxes. Yellow dots: experimental data obtained on semi−crystalline PHBV samples (experimental permeability values are estimated as P=DS for the PHBV8 sample, directly from permeation tests for the PHBV25 sample [19]). The dashed line is obtained through the linear interpolation of the simulated values reported.

Finally, we computed the values of permselectivity, estimated as the product between solubility and diffusivity contributions, and compared them with experimental data for PHBV8 and PHBV25, as shown in Table S1 and Figure 9. Additionally, the result obtained with a linear interpolation of the simulated permselectivity data is reported with a dashed line in Figure 9.

Based on such estimations, PHBV100 is the most promising candidate in terms of separation capabilities for the CO_2_/CH_4_ gas pair, as the permselectivity is higher than 50, an order of magnitude higher than the same value in PHBV0.

It must be pointed out that these results can be regarded as an order of magnitude estimates in light of the approximations regarding the semi-crystalline nature of these materials discussed previously. However, such predictions are useful to focus on the most promising candidates for a specific application.

## 4. Conclusions

In this work, MD simulations were applied to study the effect of composition on the separation properties of PHBV copolymers beyond current experimental knowledge. PHBVs were identified in previous works as promising sustainable alternatives to traditional membrane materials with an interesting sorption-driven CO_2_-selective nature. In this work, we focused on the CO_2_/CH_4_ separation performance of PHBV copolymers with increasing content of HV. The aim is that of providing hints about the optimal biopolymer formulation with target properties for specific gas separations, accelerating the introduction of sustainable materials to the market by reducing the experimental effort required.

The polymer amorphous molecular model and force field were validated using pure-polymer density and solubility parameter values from the literature for the amorphous state, as well as X-ray diffraction data. The density and solubility parameters of the copolymers decrease with the HV fraction, while the FFV increases due to the longer side chain of this monomer, which disrupts the chain packing. Random copolymers have a less ordered structure than pure homopolymers. The polymer-gas systems were simulated to extract the sorption and diffusion behaviour as a function of HV content and compared to experimental sorption and diffusion data on commercial semi-crystalline copolymers containing 8% and 25% of HV. This comparison is complicated by the semi-crystalline nature of the polymers inspected experimentally.

It is known that PHAs have a limited diffusivity–selectivity but a good solubility–selectivity for CO_2_, due to the interactions of this molecule with the ester groups of the polymer, which were investigated and explained in this work via the simulated RDFs. CO_2_ molecules show a preferential positioning around carbonyl polymer groups, indicating strong interactions which may enhance sorption but slow down diffusion with respect to CH_4_. The simulations can represent satisfactorily the CO_2_/CH_4_ solubility–selectivity value, which is particularly interesting in the case of these copolymers, whose CO_2_ capture performance relies mainly on the solubility contribution. The simulation of the diffusion selectivity of the amorphous systems overpredicts the experimental values obtained on semi-crystalline PHBV8. In general, gas-polymer systems show less regular trends with HV content than the pure polymer ones, because such monomer has opposite effects on the polymer FFV and solubility parameter δ.

However, the highest value of permselectivity is that of the pure homopolymer PHBV 100. This trend is consistent with previous experimental works that indicate CO_2_ solubility increases with HV content.

Although still limited in its accuracy, molecular simulation represents a tool to explore the performance of materials not available experimentally and to provide fundamental insight into molecular-scale behaviour. In part 2 of this series of papers, we will explore the use of a macroscopic modelling tool based on an equation of state and compare its performance with the one of the molecular simulations inspected in this work.

## Data Availability

The data presented in this study are available on request from the corresponding author.

## Supplementary Materials

The following supporting information can be downloaded at: https://www.mdpi.com/article/10.3390/polym15071805/s1, Section S1. Differential Scanning Calorimetry (DSC); Section S2. Method for the calculation of diffusivity from a sorption test; Section S3. Method for the calculation of diffusivity from Molecular Dynamics simulations; Figure S1. Representative data for MSD calculation from MD simulations. The arrow indicates the data portion used for calculations; Section S4. Radial Distribution Functions; Figure S2. Atom types present in polymeric structures; Figure S3. Radial distribution functions for intermolecolar interactions between different atom types and C-CO2 (a,c,e,g,k,n) and C-CH4 (b,d,f,h,m,o) in PHBB0, PHBV8, PHBV60, and PHBV100; Section S5. Free Fractional Volume and Accessible Surface Area; Figure S4. Free Fractional Volume (a) and Accessible Surface Area (b) for PHBV copolymers as function of HV molar percentage, calculated using penetrant sphere of 0 Å; Table S1. Solubility coefficient, Diffusivity, and selectivities for the CO2/CH4 gas pair in PHBV copolymers [17,19,37,40,47,51,52,65,66].
