# Supplementary material for: Modelling across Multiple Scales to Design Biopolymer Membranes for Sustainable Gas Separations: 1—Atomistic Approach
Source: Polymers (Basel). 2023 Apr 6;15(7):1805. doi: 10.3390/polym15071805 (PMC10097394; doi:10.3390/polym15071805)
Supplement: Supplementary file 1 [file polymers-15-01805-s001.zip › polymers-2279547-supplementary.pdf]

Supporting Information

Modelling across multiple scales to design biopolymer membranes for sustainable gas separations: 1. Atomistic approach

Kseniya Papchenko[1], Eleonora Ricci[2,#] and Maria Grazia De Angelis[1,3,*]

[1] Institute for Materials and Processes, School of Engineering, University of Edinburgh, Sanderson Building, Robert Stevenson Road, EH9 3FB, UK

[2] Department of Civil, Chemical Environmental and Materials Engineering, DICAM, University of Bologna, Via Terracini 28, 40131 Bologna, Italy

[3] National Interuniversity Consortium of Materials Science and Technology INSTM, Via G. Giusti, Firenze, 58100, Italy

[#] Current address: National Centre for Scientific Research "Demokritos", Athens, Greece

*Correspondence: grazia.deangelis@ed.ac.uk

Section S1. Differential Scanning Calorimetry (DSC)

Thermal properties of the commercial PHBV sample were evaluated by differential scanning calorimetry (DSC, Q10, TA Instruments). The complete procedure is described elsewhere [1]. The degree of crystallinity *(X_c)* was calculated as follows:

$$X_c = \Delta\hat{H}_m / \Delta\hat{H}_m^0 \qquad \text{(S1)}$$

where $\Delta\hat{H}_m$ is the melting enthalpy calculated from the endothermic peak recorded during the heating, while $\Delta\hat{H}_m^0$ is the heat of melting of purely crystalline PHB, equal to 146 J g^{-1} [2,3]. The use of such value is justified, as PHBV with less than 40 mol% HV units presents P(3HB) crystalline lattice with HV units partially excluded from it [4]. The crystallinity of the sample was determined to be equal to 42 ± 1% through DSC analysis.

Section S2. Method for the calculation of diffusivity from a sorption test.

The expression for the mass uptake as a function of time in step i, M_t^i, for the mass sorption in a limited volume, where the variation of the interfacial concentration is due to mass sorption in the membrane, is given by [5] :

$$\frac{M_t^i - M_0^i}{M_\infty^i - M_0^i} = 1 - \sum_{n=1}^{\infty} \frac{2\alpha(1+\alpha)}{1+\alpha+\alpha^2 q_n^2} \exp\left(-\mathcal{D}\frac{q_n^2 t}{l^2}\right) \qquad \text{(S2)}$$

where M_0^i and M_∞^i are the initial and final mass uptake in step i, respectively, and α is the ratio between the volume of solution and that of the membrane, corrected for the partition coefficient of vapor between the gaseous phase and the polymer, l is the semithickness of the membrane, while q_n variables are the positive, nonzero, solutions of the equation: $tg(q_n) = -\alpha q_n$. By fitting the experimental data of mass uptake versus time to the aforementioned equation, one obtains the average diffusivity value within the concentration interval inspected in the differential sorption step.

Section S3. Method for the calculation of diffusivity from Molecular Dynamics simulations.

Mean-squared displacement of gas molecules along the NVE trajectories can be used to calculated the self-diffusion coefficient through the Einstein relation, once the Fickian regime is reached [6,7]. The logarithm of MSD can be plotted against the logarithm of time and the Fickian regime is identified by the portion of the graph, characterized by a slope equal to 1 of log(MSD) vs log(t). The slope of MSD vs t in such region is then used for the Diffusivity coefficient calculation. Self-diffusivities are a good approximation of binary diffusivities in the case of an infinitely dilute system or when the sorption isotherm is linear [8].

Figure S1 shows an example of the MDS data used for diffusivity coefficient calculation in case of CO_2 in PHBV100 system, with average value extracted from three independent configurations.

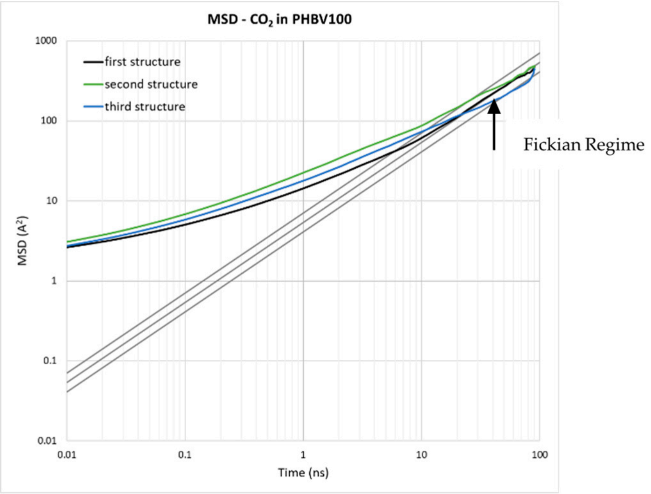

Figure S1. Representative data for MSD calculation from MD simulations. The arrow indicates the data portion used for calculations.

Section S4. Radial Distribution Functions

Radial distribution functions can be used to describe molecular packing in macromolecular systems. Here we show intermolecular interactions between different atom types in PHBB0, PHBV8, PHBV60, and PHBV100 and center of mass of gas molecules, well represented by carbon atoms, C-CO_2 and C-CH_4. Figure S2 provides a legend to atom description in hydroxybutyrate and hydroxyvalerate monomers. Figure S3 shows intermolecular interactions between different atoms in four polymeric structures and C-CO_2 (left side) or C-CH_4 (right side). To facilitate the reader, atom type numbers are reported also on Figure S3.

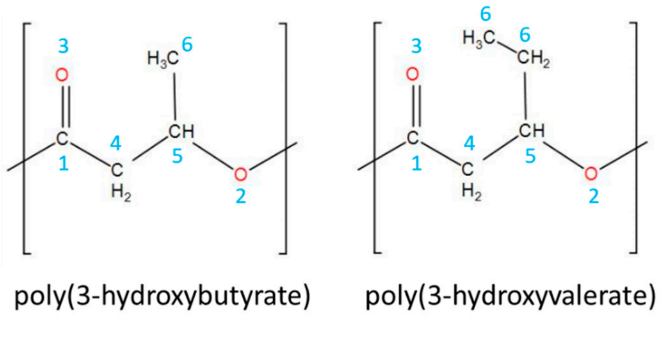

Figure S2. Atom types present in polymeric structures.

1 – Carbonyl C, 2 – Ester O, 3 – Carbonyl O,
4 – Backbone C, 5 – Backbone C, 6 – Side chain C.

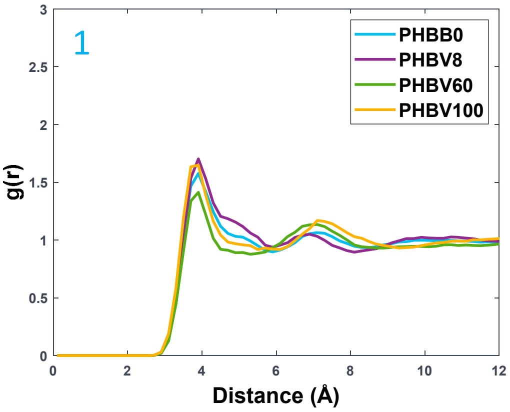
1
PHBB0
PHBV8
PHBV60
PHBV100
g(r)
Distance (Å)

(a)

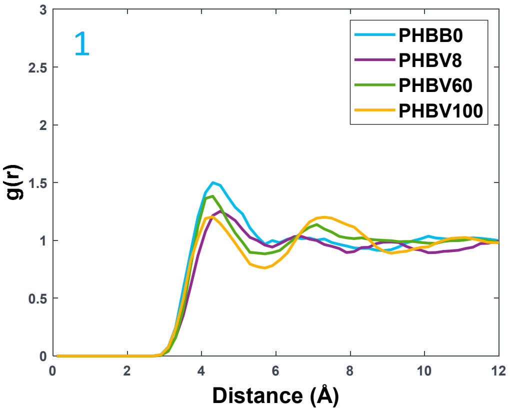
1
PHBB0
PHBV8
PHBV60
PHBV100
g(r)
Distance (Å)

(b)

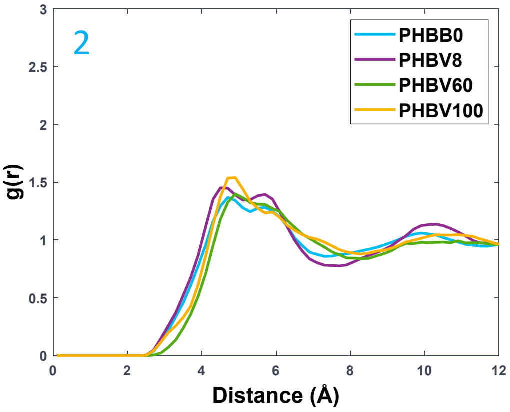
2
PHBB0
PHBV8
PHBV60
PHBV100
g(r)
Distance (Å)

(c)

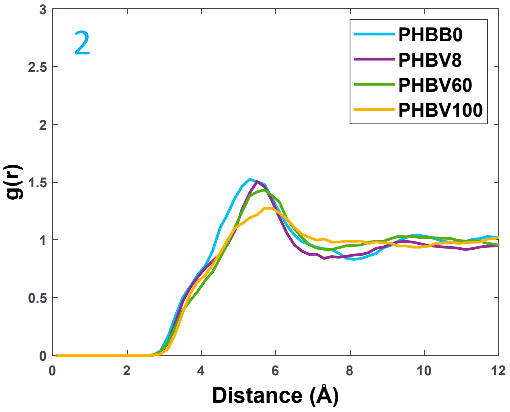
2
PHBB0
PHBV8
PHBV60
PHBV100
g(r)
Distance (Å)

(d)

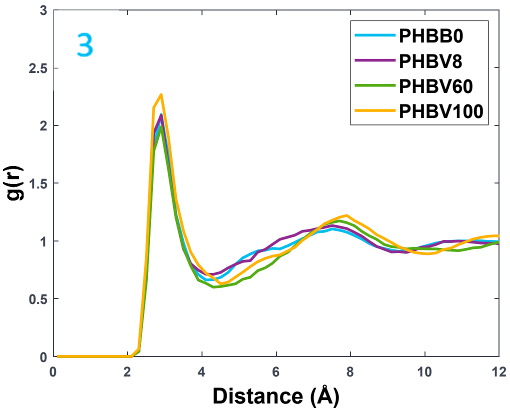
3
PHBB0
PHBV8
PHBV60
PHBV100
g(r)
Distance (Å)

(e)

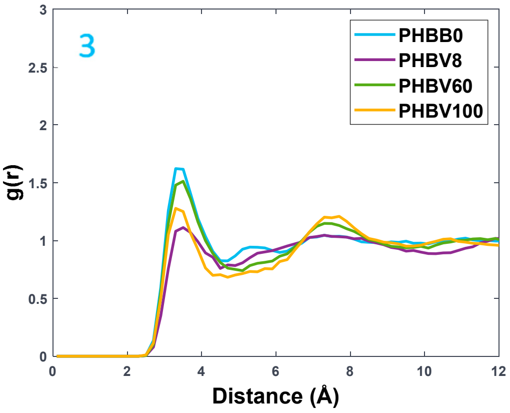
3
PHBB0
PHBV8
PHBV60
PHBV100
g(r)
Distance (Å)

(f)

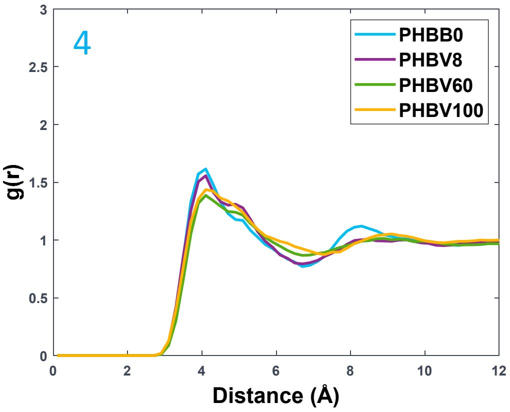

(g)

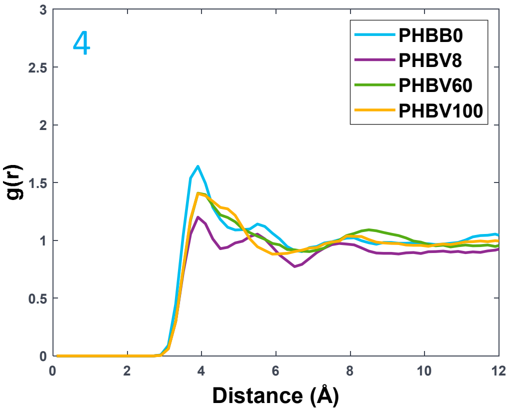

(h)

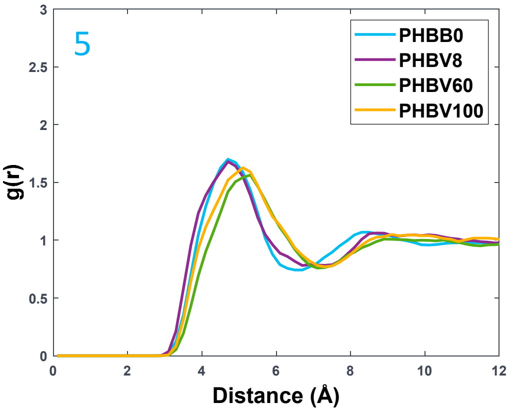

(k)

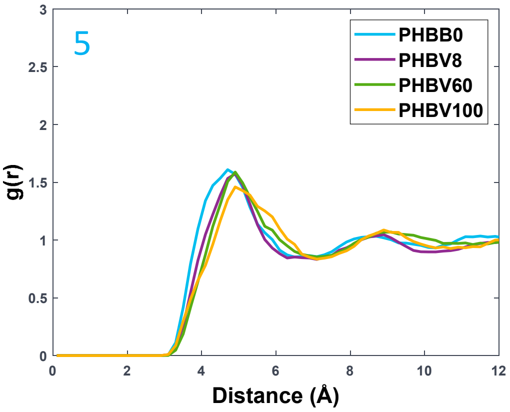

(m)

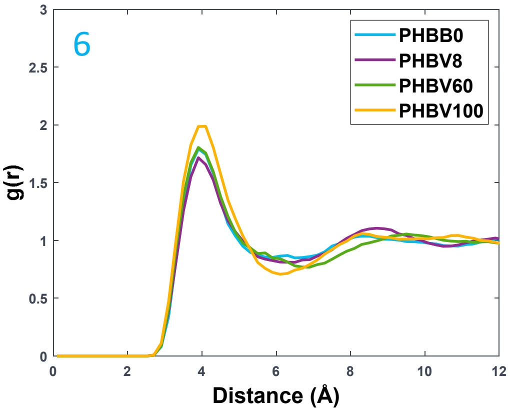

(n)

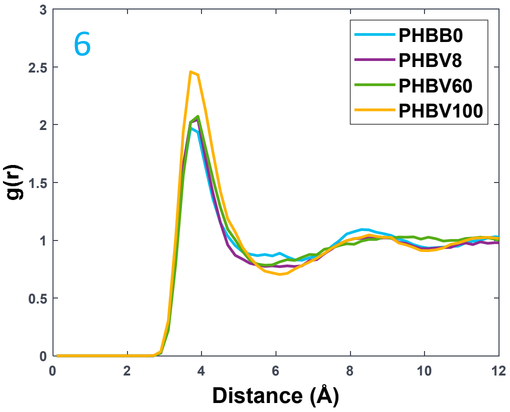

(o)

Figure S3. Radial distribution functions for intermolecolar interactions between different atom types and C-CO_2 (a,c,e,g,k,n) and C-CH_4 (b,d,f,h,m,o) in PHBB0, PHBV8, PHBV60, and PHBV100.

Section S5. Free Fractional Volume and Accessible Surface Area

Fractional free volume (FFV) and accessible surface area are two structural parameters that can give additional information on molecular packing of the structure. FFV and accessible surface area of different copolymers were evaluated for the solvent sphere of 0 Å using the MAPS plug-in [9].

FFV gives a measure of the chain packing efficiency and can be estimated from MD runs as follows:

$$FFV = \frac{V_0 - V_c}{V_0} \qquad (S1)$$

where V_0 is the volume of MD cell and V_c is the total volume occupied by the polymer segments.

The accessible surface area is defines as the external surface area of polymeric segments in contact with the penetrant molecule, and thus is closely related to the FFV.

The values are reported in Figure S4.

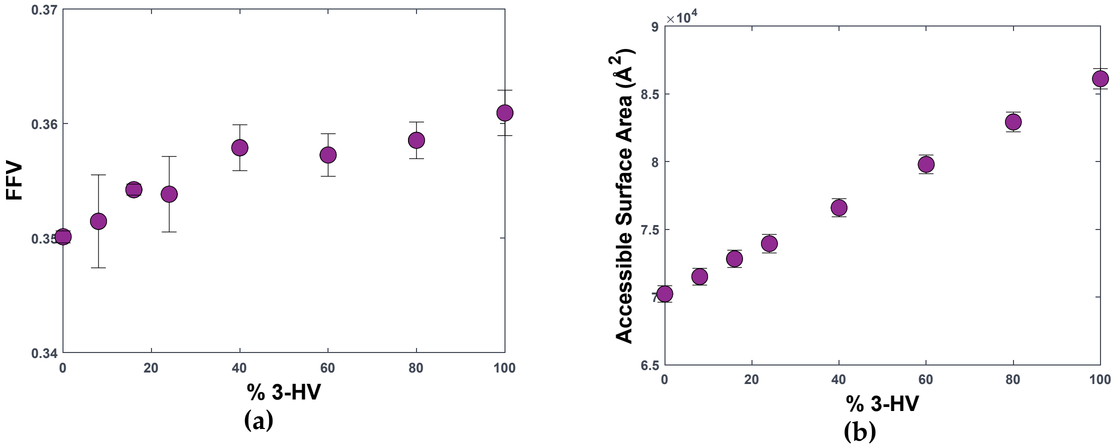

Figure S4. Free Fractional Volume (a) and Accessible Surface Area (b) for PHBV copolymers as function of HV molar percentage, calculated using penetrant sphere of 0 Å.

Table S1. Solubility coefficient, Diffusivity, and selectivities for the CO_2/CH_4 gas pair in PHBV copolymers.

	**PHBV0 (sim.)**	**PHBV8 (sim.)**	**PHBV8 (exp.)**	**PHBV25 (exp.)** [1]	**PHBV60 (sim.)**	**PHBV100 (sim.)**
			S_i^{am} **× 10**^{3} [kg/(kg$_{am.pol.}$ MPa)]			
CO_2	15 ± 8	12 ± 3	21 ± 1	37 ± 5	34 ± 12	30 ± 4
CH_4	1.9 ± 0.4	0.5 ± 0.3	1.5 ± 0.4	0.9 ± 0.1	1.9 ± 0.3	1.2 ± 0.4
			**Selectivity (molar basis)**			
$\alpha_S^{CO_2/CH_4}$	2.8 ± 1.6	8.7 ± 5.7	5.1 ± 1.4	14 ± 2.4	6.5 ± 2.5	9.3 ± 3.3
$\alpha_D^{CO_2/CH_4}$	1.1 ± 0.5	3.4 ± 1.4	0.6 ± 0.2	1.9 ± 0.3	0.3 ± 0.1	5.5 ± 2.3
α^{CO_2/CH_4}	3.2 ± 2.3	29 ± 23	3.1 ± 1.3	27 ± 6	1.6 ± 0.9	51 ± 29

References

[1] K. Papchenko, M. Degli Esposti, M. Minelli, P. Fabbri, D. Morselli, M.G. De Angelis, New sustainable routes for gas separation membranes: The properties of poly(hydroxybutyrate-co-hydroxyvalerate) cast from green solvents, Journal of Membrane Science. 660 (2022) 120847. https://doi.org/10.1016/j.memsci.2022.120847.

[2] N. Follain, C. Chappey, E. Dargent, F. Chivrac, R. Crétois, S. Marais, Structure and Barrier Properties of Biodegradable Polyhydroxyalkanoate Films, J. Phys. Chem. C. 118 (2014) 6165–6177. https://doi.org/10.1021/jp408150k.

[3] P.J. Barham, A. Keller, E.L. Otun, P.A. Holmes, Crystallization and morphology of a bacterial thermoplastic: poly-3-hydroxybutyrate, J Mater Sci. 19 (1984) 2781–2794. https://doi.org/10.1007/BF01026954.

[4] N. Kamiya, M. Sakurai, Y. Inoue, R. Chujo, Isomorphic behavior of random copolymers: thermodynamic analysis of cocrystallization of poly(3-hydroxybutyrate-co-3-hydroxyvalerate), Macromolecules. 24 (1991) 3888–3892. https://doi.org/10.1021/ma00013a023.

[5] J. Crank, E.P.J. Crank, The Mathematics of Diffusion, Clarendon Press, 1979.

[6] Frenkel, D.; Smit, B. Understanding Molecular Simulation: From Algorithms to Applications; Elsevier: 2001., n.d.

[7] M.P. Allen, D.J. Tildesley, D.J. Tildesley, Computer simulation of liquids, Reprint, Clarendon Press, Oxford, 1990.

[8] Y. Yampolskii, I. Pinnau, B. Freeman, eds., Materials Science of Membranes for Gas and Vapor Separation: Freeman/Materials Science of Membranes for Gas and Vapor Separation, John Wiley & Sons, Ltd, Chichester, UK, 2006. https://doi.org/10.1002/047002903X.

[9] https://www.scienomics.com; MAPS Platform.
